# COVID-19 and renal allograft rejection: insight from controlled and non-controlled studies

**DOI:** 10.1080/0886022X.2024.2336126

**Published:** 2024-04-16

**Authors:** Ahmed Daoud, Karim Soliman, Maria Aurora Posadas Salas, Genta Uehara, Sakshi Vaishnav, Wisit Cheungpasitporn, Michael J. Casey

**Affiliations:** a Medical University of South Carolina, Charleston, SC, USA; b Division of Nephrology, Cairo University Medical School, Cairo, Egypt; c Medical Services, Ralph H. Johnson VA Medical Center, Charleston, SC, USA; d Division of Nephrology and Hypertension, Mayo Clinic, Rochester, MN, USA

**Keywords:** COVID, renal, kidney, transplant, rejection

## Abstract

**Aim:**

Kidney transplant recipients (KTRs), due to their immunosuppressed status, are potentially more susceptible to both the severe effects of COVID-19 and complications in their transplanted organ. The aim of this study is to investigate whether COVID-19 infection increases the risk of rejection in kidney transplant recipients (KTRs).

**Methods:**

This study involved a detailed literature review, conducted using PubMed, with the search being completed by September 7th, 2023. The search strategy incorporated a combination of relevant keywords: ‘COVID’, ‘Renal’, ‘Kidney’, ‘Transplant’, and ‘Rejection’. The results from controlled and uncontrolled studies were separately collated and analyzed.

**Results:**

A total of 11 studies were identified, encompassing 1,179 patients. Among these, two controlled studies reported the incidence of rejection in KTRs infected with COVID-19. Pooling data from these studies revealed no significant statistical correlation between COVID-19 infection and biopsy-proven rejection (*p* = 0.26). In addition, nine non-controlled studies were found, with rejection incidences ranging from 0% to 66.7%. The majority of these studies (eight out of nine) had small sample sizes, ranging from 3 to 75 KTRs, while the largest included 372 KTRs. The combined rejection rate across these studies was calculated to be 11.8%.

**Conclusion:**

In conclusion, the limited number of published controlled studies revealed no statistically significant association between COVID-19 infection and biopsy-proven rejection among KTRs. However, the broader analysis of non-controlled studies showed a variable rejection incidence with a pooled rejection rate of 11.8%. There is insufficient high-quality data to explore the association of COVID-19 infection and rejection.

It is well known that Coronavirus disease 2019 (COVID-19) caused by severe acute respiratory syndrome coronavirus 2 (SARS COV-2) can be severe in immunosuppressed patients, particularly kidney transplant recipients (KTRs). KTRs are maintained on lifelong immunosuppression to reduce the risk of cellular and antibody mediated rejection. The optimum management of immunosuppression in KTRs infected with COVID-19 remains unclear [1]. Nevertheless, a prevailing clinical approach involves the reduction of immunosuppressive therapy in cases of symptomatic COVID-19 infection among KTRs. This scenario presents several pivotal questions: Does SARS-CoV-2 infection elevate the rejection risk in KTRs? Could the diminution of immunosuppression therapy heighten the risk of rejection? What insights does the extant literature provide on this subject?

To answer these questions, we conducted a detailed literature review, conducted using PubMed as the primary database, with the search being completed by September 7th, 2023. The search strategy incorporated a combination of keywords: ‘COVID’, ‘Renal’, ‘Kidney’, ‘Transplant’, and ‘Rejection’. This process involved the examination of 232 manuscripts, leading to the exclusion of:

- 207 papers with unrelated results that did not report rejection incidence in COVID-infected KTRs,
- 12 case reports,
- 1 case series, and
- 1 review article.

Ultimately, we identified eleven studies that specifically reported on the incidence of rejection in COVID-infected KTRs. Notably, two of these studies incorporated control groups (Figure 1).

**Figure 1.**
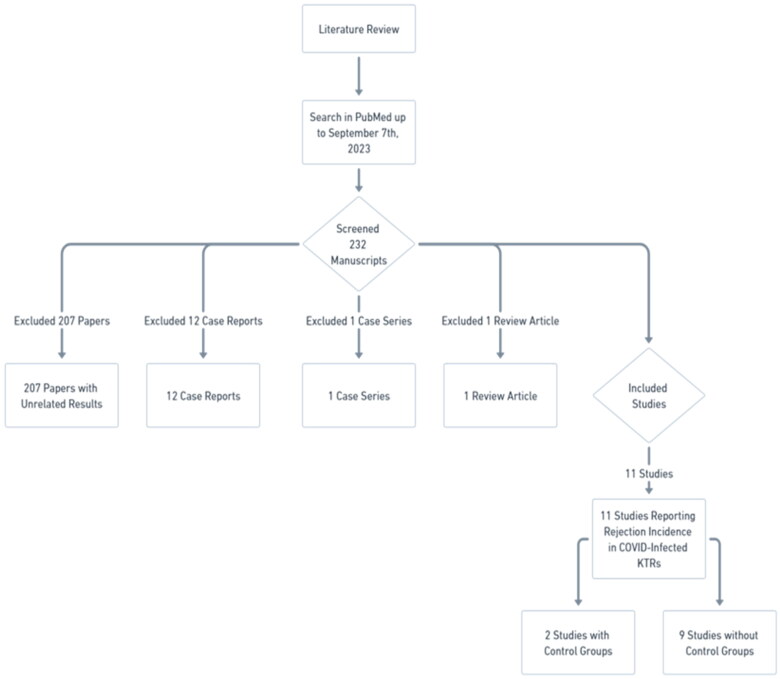
Literature search.

- Asti et al. [2] included 201 KTRs from Northern Italy. Out of 29 SARS-CoV-2 IgG positive patients due to COVID-infection, 2 patients (6.9%) developed biopsy proven rejection (one acute cellular rejection (ACR) and the other chronic rejection). Out of 172 SARS-CoV-2 IgG negative patients, 10 patients (5.8%) developed biopsy proven rejection (six ACRs, two antibody mediated rejections (AMR), and two chronic rejections). No significant association was found between rejection incidence and SARS-CoV-2 IgG positivity (P = 0.69).
- Oto et al. [3] performed a multicenter study in Turkey which included 944 KTRs, where 523 patients were infected with COVID-19, while 421 were not. Diagnosis of COVID-19 was confirmed by a nasopharyngeal swab positive reverse transcriptase polymerase chain reaction test. Incidence of biopsy proven acute rejection were low among both groups with no statistically significant difference (6 out of 523 (1.1%) COVID-19 patients and 4 out of 421 (0.95%) non-COVID-19 patients). Duration of follow up was 3 months after COVID infection.

Subsequent to pooling data from both studies (Table 1), we discerned no statistically significant relationship between COVID-19 infection and biopsy-proven rejection (*p* = 0.26). However, given the paucity of controlled studies in this domain—predominantly represented by the study by Oto et al.—interpretive caution is advised.

**Table 1.** Incidence of rejection among COVID19 and non-COVID patients (controlled studies).

	Rejection--yes	Rejection--no	Total
COVID-19 positive	8	544	552
COVID-19 negative	14	579	593
Total	22	1123	1145

Additionally, an examination of nine non-controlled studies reporting on the incidence of rejection among COVID-19 infected KTRs revealed a wide variance, ranging from 0% to 66.7%. (See Supplementary material, Table S1) The majority of these studies included between 3 to 75 KTRs, with the largest study encompassing 372 KTRs [4]. The aggregated incidence of rejection in these non-controlled studies was 11.8%. It is crucial to approach these findings with circumspection due to the uncontrolled nature of these studies. Interestingly, all but one of the non-controlled studies reported biopsy-proven rejections. Excluding the study by Kute et al. [4], there were forty cases of biopsy-proven rejections, which can be categorized as follows:

- Nineteen of forty (47.5%) are AMR,
- Sixteen of forty (40%) are cellular rejections (including borderline), and
- Five of forty (12.5%) are mixed cellular and antibody mediated rejections.

Vinson et al. performed a large retrospective study including 18121 solid organ transplant (SOT) recipients. COVID-19 infection was associated with a higher risk of rejection. However, no data about rejection in KTRs was reported separately by the authors [5]. A small study performed by Ali et al. showed that none of five KTRs, receiving kidneys from donors who tested positive for COVID-19 by polymerase chain reaction developed any episodes of rejection [6].

There are many variables that may impact rejection risk in COVID-19 infected KTRs. These variables include the severity of COVID-19 infection, extent and duration of immunosuppression reduction, induction regimen, Human leukocyte antigen (HLA) mismatch, Calculated Panel Reactive Antibody (CPRA), prior episodes of rejection, presence of donor specific antibodies (DSA) and the trough levels of calcineurin inhibitors (CNIs) and mammalian target of rapamycin (mTOR) inhibitors. The data in literature about the association between these factors and rejection risk in COVID-19 infected KTRs remain scarce. According to Kute et al. [4], absence of Oxygen requirement during COVID-19 compared to Oxygen need and thymoglobulin induction were associated with a lower risk of rejection. On the other hand, HLA-DR mismatch had the highest risk of acute rejection. The use of steroids had no statistically significant effect on the risk of rejection. The study performed by Bae et al. showed that the use of thymoglobulin induction decreased in favor of basiliximab and no induction during the COVID-19 era. This may have led to higher rates of rejection among KTRs during the pandemic [7].

In summary, it is imperative to acknowledge that the current corpus of high-quality data remains insufficient to conclusively ascertain the relationship between COVID-19 infection and renal allograft rejection. Our literature review yielded only a limited number of studies pertinent to this inquiry, specifically two controlled and nine non-controlled studies. These studies are characterized by a relatively small cohort size and exhibit considerable variability in their outcomes. Moreover, the landscape of our understanding is further complicated by the absence of comprehensive data on several critical factors. These include the dynamics of immunosuppression reduction in the context of COVID-19, the evolving protocols surrounding COVID vaccination, and the integration of advanced diagnostic methodologies such as polymerase chain reaction (PCR) testing.

Given these gaps in our knowledge, it is evident that there is a pronounced need for more rigorously controlled studies. These studies should not only focus on the direct relationship between COVID-19 infection and rejection rates in KTRs but also incorporate an analysis of the aforementioned additional variables. Such expansive research endeavors are essential to determine whether COVID-19 infection correlates with an elevated risk of rejection in KTRs. The acquisition of this knowledge is vital for the formulation of optimized management strategies for this vulnerable patient population.

## Supplementary Material

Supplemental Material
