# Supplementary material for: COVID-19 and renal allograft rejection: insight from controlled and non-controlled studies
Source: Ren Fail. 2024 Apr 16;46(1):2336126. doi: 10.1080/0886022X.2024.2336126 (PMC11022909; doi:10.1080/0886022X.2024.2336126)
Supplement: Supplemental Material [file IRNF_A_2336126_SM9325.docx]

**COVID & Rejection: Supplementary material**

**Table 1. Incidence of Rejection Among COVID19 Patients (Non-Controlled Studies)**

| Study | Number of patients | Number of patients with rejection | Incidence of Rejection |
| --- | --- | --- | --- |
| Vásquez-Jiménez et al (1) | 20 | 14  Borderline 2, Chronic active TCMR 1, Active AMR 3, Mixed AMR/ TCMR 4, Chronic active AMR 4) | 60% |
| Kute et al (2) | 372 | 34 (23 rejections were biopsy proven) | 9.1% |
| Daniel et al (3) | 18 | 5 (4 TCMR, 1 AMR + Borderline rejections) | 27.78% |
| Kute et al (4) | 64 | 6 (3 ACR + 3 AMR) | 9.375% |
| Akilesh et al (5) | 3 | 2 (AMR) | 66.667% |
| Felldin et al (6) | 31 | 0 | 0% |
| Basic-Jukic et al (7) | 6 | 3 (1 borderline ACR + 2 chronic AMR) | 50% |
| Kute et al (8) | 75 | 5 (AMR = 2, TCMR = 3) | 6.6% |
| Kute et al (9) | 38 ABOiKTx | 5 (3 AMR + 2 ACR) | 13.1% |
| Total | 627 | 74 | 11.8% |

**TCMR = T-cell Mediated Rejection, AMR = Antibody Mediated Rejection, ACR = Acute Cellular Rejection, ABOiKTx = ABO-incompatible kidney transplant**

**Supplemental Bibliography:**

1--Vásquez-Jiménez E, Moguel-González B, Soto-Abraham V, Flores-Gama C. Risk of acute rejection in kidney transplant recipients after COVID-19. J Nephrol. 2022 Jan;35(1):367-369. doi: 10.1007/s40620-021-01192-x. Epub 2021 Nov 17. PMID: 34787799; PMCID: PMC8596849.

2-Kute VB, Ray DS, Aziz F, Godara SM, Hegde U, KumarBT A, Bhalla AK, Yadav DK, Singh S, Pathak V, Dalal S, Bahadur MM, Anandh U, Abraham M A, Siddini V, Das SS, Thukral S, Krishnakumar A, Sharma A, Kher V, Bansal SB, Shingare A, Narayanan R, Patel H, Gulati S, Kakde S, Bansal D, Guleria S, Khullar D, Gumber MR, Varyani U, Guditi S, Khetan P, Dave R, Mishra VV, Tullius SG, Chauhan S, Meshram HS. Management strategies and outcomes in renal transplant recipients recovering from COVID-19: A retrospective, multicentre, cohort study. EClinicalMedicine. 2022 Apr;46:101359. doi: 10.1016/j.eclinm.2022.101359. Epub 2022 Mar 25. PMID: 35350707; PMCID: PMC8948372.

3-Daniel E, Sekulic M, Kudose S, Kubin C, Ye X, Shayan K, Patel A, Cohen DJ, E Ratner L, Santoriello D, Barry Stokes M, Markowitz GS, Pereira MR, D'Agati VD, Batal I. Kidney allograft biopsy findings after COVID-19. Am J Transplant. 2021 Dec;21(12):4032-4042. doi: 10.1111/ajt.16804. Epub 2021 Sep 3. PMID: 34403563; PMCID: PMC8441660.

4-Kute VB, Aziz F, Abraham A, Ray DS, Pathak V, Siddini V, Hegde U, Chauhan S, Meshram HS, Group I. Outcomes of Living Donor Kidney Transplant After SARS-CoV-2 Infection in Both the Donor and the Recipient: A Multicenter Study. Exp Clin Transplant. 2022 Oct;20(10):908-916. doi: 10.6002/ect.2022.0205. PMID: 36409050.

5-Akilesh S, Nast CC, Yamashita M, Henriksen K, Charu V, Troxell ML, Kambham N, Bracamonte E, Houghton D, Ahmed NI, Chong CC, Thajudeen B, Rehman S, Khoury F, Zuckerman JE, Gitomer J, Raguram PC, Mujeeb S, Schwarze U, Shannon MB, De Castro I, Alpers CE, Najafian B, Nicosia RF, Andeen NK, Smith KD. Multicenter Clinicopathologic Correlation of Kidney Biopsies Performed in COVID-19 Patients Presenting With Acute Kidney Injury or Proteinuria. Am J Kidney Dis. 2021 Jan;77(1):82-93.e1. doi: 10.1053/j.ajkd.2020.10.001. Epub 2020 Oct 10. PMID: 33045255; PMCID: PMC7546949.

6-Felldin M, Søfteland JM, Magnusson J, Ekberg J, Karason K, Schult A, Larsson H, Oltean M, Friman V. Initial Report From a Swedish High-volume Transplant Center After the First Wave of the COVID-19 Pandemic. Transplantation. 2021 Jan 1;105(1):108-114. doi: 10.1097/TP.0000000000003436. PMID: 32826796.

7-Basic-Jukic N, Coric M, Bulimbasic S, Dika Z, Juric I, Furic-Cunko V, Katalinic L, Kos J, Fistrek M, Kastelan Z, Jelakovic B. Histopathologic findings on indication renal allograft biopsies after recovery from acute COVID-19. Clin Transplant. 2021 Dec;35(12):e14486. doi: 10.1111/ctr.14486. Epub 2021 Oct 1. PMID: 34532893; PMCID: PMC8646844.

8-Kute VB, Ray DS, Yadav DK, Pathak V, Bhalla AK, Godara S, Kumar A, Guleria S, Khullar D, Thukral S, Mondal RRS, Jain M, Jha PK, Hegde U, Abraham M A, Dalal S, Patel H, Bahadur MM, Shingare A, Sharma A, Kumar Sharma R, Anandh U, Gulati S, Gumber M, Siddini V, Deshpande R, Kaswan K, Varyani U, Kakde S, Kenwar DB, Shankar Meshram H, Kher V. A Multicenter Cohort Study From India of 75 Kidney Transplants in Recipients Recovered After COVID-19. Transplantation. 2021 Jul 1;105(7):1423-1432. doi: 10.1097/TP.0000000000003740. PMID: 33724246.

9-Kute V, Ray DS, Dalal S, Hegde U, Godara S, Pathak V, Bahadur MM, Khullar D, Guleria S, Vishwanath S, Singhare A, Yadav D, Bansal SB, Chauhan S, Meshram HS. A Multicenter Cohort Study From India of ABO-Incompatible Kidney Transplantation in Post-COVID-19 Patients. Transplant Proc. 2022 Dec;54(10):2652-2657. doi: 10.1016/j.transproceed.2022.07.002. Epub 2022 Jul 21. PMID: 35995711; PMCID: PMC9300716.
